# Stability and Hopf Bifurcation of a Vector-Borne Disease Model with Saturated Infection Rate and Reinfection

**DOI:** 10.1155/2019/1352698

**Published:** 2019-06-09

**Authors:** Zhixing Hu, Shanshan Yin, Hui Wang

**Affiliations:** School of Mathematics and Physics, University of Science and Technology Beijing, Beijing 100083, China

## Abstract

This paper established a delayed vector-borne disease model with saturated infection rate and cure rate. First of all, according to the basic reproductive number *R*_0_, we determined the disease-free equilibrium *E*_0_ and the endemic equilibrium *E*_1_. Through the analysis of the characteristic equation, we consider the stability of two equilibriums. Furthermore, the effect on the stability of the endemic equilibrium *E*_1_ by delay was studied, the existence of Hopf bifurcations of this system in *E*_1_ was analyzed, and the length of delay to preserve stability was estimated. The direction and stability of the Hopf bifurcation were also been determined. Finally, we performed some numerical simulation to illustrate our main results.

## 1. Introduction

Malaria is a vector-borne infectious disease [1], caused by parasites. It is popular in 102 countries and regions, especially in some countries in Africa, southeast Asia, and South America. In the 30s of this century, malaria spread throughout the country. Clinical symptoms and signs of this disease, such as typical periodic onset of malaria, secondary anemia, and spleen, can cause serious consequences, including dangerous malaria, malarial kidney disease, and black urine fever.

The main way of transmission of malaria is the bite of an infected female anopheline mosquito. The mosquitoes would also be infected when uninfected mosquitoes bite infected people, and this transmission process has an incubation period [2]. The important feature of malaria is that the recovered immune system may establish immune memory for such antigens. It is this characteristic that greatly reduces the spread of malaria [3, 4]. Immune process is slow and, however, takes years or even decades [5]. As time goes by, the immune system gradually weakens, and at this time, reinfection likely occurs; therefore, considering the function of delay and immune system is necessary in the study of malaria.

For the vector-borne diseases such as malaria, a large number of mathematical models have been created [2, 6, 7, 8, 9], most of which consider the local immunity and delay of the spread of malaria in the crowd. Different time delay has been used to describe the latent period in the course of disease transmission [7, 8, 9]. Local stability conditions for the equilibrium of a model with two time delays have been considered by Wan and Cui [8]. The global stability of the equilibrium has been studied for a vector-borne disease model with distributed delay by Cai et al. [10].

Based on the above model, this paper considers a delayed vector-borne model with saturated infection rate and partial immunity to reinfection. We prove that the stability of this system can be changed by time delay and produce Hopf bifurcation, calculating the length of delay to preserve stability. Using the center manifold theorem [11] and norm theory, we determine the stability and bifurcation direction.

## 2. Model Formulation

*N*_1_(*t*) represented as the host population at time *t* is divided into three subclasses: the susceptible *S*(*t*), the infected *I*(*t*), and the recovered *R*(*t*). *N*_2_(*t*) represented as the vector population at time *t* is divided into two subclasses: the susceptible *T*(*t*) and the infected *V*(*t*). The Hopf bifurcation was determined in a model with direct infection and delay by Wei et al. [9]. The mathematical formulation still needs improvements. We consider an improved model as follows:

(1) $$\begin{array}{c} \left\{\begin{array}{c} \frac{dS}{dt}=\Lambda_{1}-\frac{b\beta_{1}S\left(t\right)V\left(t\right)}{1+\alpha V\left(t\right)}-\mu_{1}S\left(t\right),\\ \frac{dI}{dt}=\frac{b\beta_{1}S\left(t\right)V\left(t\right)}{1+\alpha V\left(t\right)}+\sigma b\beta_{1}R\left(t\right)V\left(t\right)-\left(\mu_{1}+\gamma \right)I\left(t\right),\\ \frac{dR}{dt}=\gamma I\left(t\right)-\sigma b\beta_{1}R\left(t\right)V\left(t\right)-\mu_{1}R\left(t\right),\\ \frac{dT}{dt}=\Lambda_{2}-b\beta_{2}T\left(t-\tau \right)I\left(t-\tau \right)-\mu_{2}T\left(t\right),\\ \frac{dV}{dt}=b\beta_{2}T\left(t-\tau \right)I\left(t-\tau \right)-\mu_{2}V\left(t\right), \end{array}\right. \end{array}$$

where Λ_1_ and Λ_2_ represent the recruitment rate of the host population and vector population, respectively. *b* represents the average number of bites per mosquito per day. The incidence rate *bβ*_1_ *S*(*t*)*V*(*t*)/1+*αV*(*t*) is the number of infections of the susceptible host caused by the infected vector, and *α* is the inhibitory effect rate caused by the infected vector. *μ*_1_ and *μ*_2_ represent the death rates of the host population and vector population, respectively. *β*_1_ is the infection rate from vector to human. *σ*(0 ≤ *σ* ≤ 1) represents the degree of partial protection for recovered people given by a primary infection, where *σ*=0 represents complete protection and *σ*=1 represents no protection. *γ* is the per capita recovery rate of the infected host population. *β*_2_ represents the infection rate from human to vector. *τ* is the time delay, representing the incubation period in the vector population; that is to say, a susceptible vector that bites an infective host at time *t* − *τ* will become infective at time *t*.

The model (1) meets the initial conditions:

(2) $$\begin{array}{c} \left\{\begin{array}{c} S\left(\theta \right)=\varphi_{1}\left(\theta \right),\\ I\left(\theta \right)=\varphi_{2}\left(\theta \right),\\ R\left(\theta \right)=\varphi_{3}\left(\theta \right),\\ T\left(\theta \right)=\varphi_{4}\left(\theta \right),\\ V\left(\theta \right)=\varphi_{5}\left(\theta \right),\\ \varphi_{i}\left(\theta \right)\geq 0,\;\left(i=1,2,3,4,5\right),\\ -\tau \leq \theta \leq 0, \end{array}\right. \end{array}$$

where (*φ*_1_(*θ*), *φ*_2_(*θ*), *φ*_3_(*θ*), *φ*_4_(*θ*), *φ*_5_(*θ*))  ∈*C*([−*τ*, 0], *R*_+_^5^) is the Banach space of continuous functions mapping the interval [−*τ*, 0] into *R*_+_^5^ with the topology of uniform convergence. The norm is defined as follows:

(3) $$\begin{array}{c} \left\|\varphi \right\|=\underset{-\tau \leq \theta \leq 0}{\sup}\left\{\left|\varphi_{1}\left(\theta \right)\right|,\left|\varphi_{2}\left(\theta \right)\right|,\left|\varphi_{3}\left(\theta \right)\right|,\left|\varphi_{4}\left(\theta \right)\right|,\left|\varphi_{5}\left(\theta \right)\right|\right\}. \end{array}$$

Based on the fundamental theory of functional differential equations [12], it is easy to show that the solution of the model (1) with the initial condition (2) is unique and is nonnegative for all *t* ≥ 0.

By (1), we know that

(4) $$\begin{array}{c} N_{k}^{\prime }\left(t\right)=\Lambda_{k}-\mu_{k}N_{k}\left(t\right),\;\left(k=1,2\right), \end{array}$$

and can solve it by using the integrating factor:

(5) $$\begin{array}{c} N_{k}\left(t\right)=N_{k}\left(0\right)\;e^{-\mu_{k}t}+\frac{\Lambda_{k}}{\mu_{k}}\left(1-e^{-\mu_{k}t}\right). \end{array}$$

That is,

(6) $$\begin{array}{c} \underset{t⟶\infty }{\lim}N_{k}\left(t\right)=\frac{\Lambda_{k}}{\mu_{k}},\;\left(k=1,2\right). \end{array}$$

Form the limiting theory of differential equation [13], we can draw that model (1) is the equivalent of the following equation:

(7) $$\begin{array}{c} \left\{\begin{array}{c} \frac{dS}{dt}=\Lambda_{1}-\frac{b\beta_{1}S\left(t\right)\;V\left(t\right)}{1+\alpha V\left(t\right)}-\mu_{1}S\left(t\right),\\ \frac{dI}{dt}=\frac{b\beta_{1}S\left(t\right)\;V\left(t\right)}{1+\alpha V\left(t\right)}+\sigma b\beta_{1}\left(\frac{\Lambda_{1}}{\mu_{1}}-S\left(t\right)-I\left(t\right)\right)V\left(t\right)-\left(\mu_{1}+\gamma \right)I\left(t\right),\\ \frac{dV}{dt}=b\beta_{2}\left(\frac{\Lambda_{2}}{\mu_{2}}-V\left(t-\tau \right)\right)I\left(t-\tau \right)-\mu_{2}V\left(t\right). \end{array}\right. \end{array}$$

Next, the model (7) can be studied in the invariant set:

(8) $$\begin{array}{c} \Omega =\left\{\left(S,I,V\right)\in R_{+}^{3}\;|\;0\leq S+I\leq \frac{\Lambda_{1}}{\mu_{1}},0\leq V\leq \frac{\Lambda_{2}}{\mu_{2}},S\geq 0,I\geq 0\right\}. \end{array}$$

Now, let us consider the existence of equilibrium.

First, it is easy to show that system (7) always has a disease-free equilibrium *E*_0_=(*S*_0_, *I*_0_, *V*_0_)=(Λ_1_/*μ*_1_, 0,0). The endemic equilibrium *E*_1_=(*S*_1_, *I*_1_, *V*_1_) satisfies the following equation:

(9) $$\begin{array}{c} \left\{\begin{array}{c} \Lambda_{1}-\frac{b\beta_{1}S_{1}V_{1}}{1+\alpha V_{1}}-\mu_{1}S_{1}=0,\\ \frac{b\beta_{1}S_{1}V_{1}}{1+\alpha V_{1}}+\sigma b\beta_{1}\left(\frac{\Lambda_{1}}{\mu_{1}}-S_{1}-I_{1}\right)V_{1}-\left(\mu_{1}+\gamma \right)I_{1}=0,\\ b\beta_{2}\left(\frac{\Lambda_{2}}{\mu_{2}}-V_{1}\right)I_{1}-\mu_{2}V_{1}=0. \end{array}\right. \end{array}$$

Form (9), we have *S*_1_=Λ_1_(1+*αV*_1_)/*bβ*_1_ *V*_1_+*μ*_1_(1+*αV*_1_) and *I*_1_=*μ*_2_ *V*_1_/*bβ*_2_((Λ_2_/*μ*_2_) − *V*_1_), where *V*_1_ satisfies the following equation:

(10) $$\begin{array}{c} P_{2}V^{2}+P_{1}V+P_{0}=0, \end{array}$$

where

(11) $$\begin{array}{c} P_{2}=\sigma b\beta_{1}\left[\Lambda_{1}b^{2}\beta_{1}\beta_{2}+\mu_{1}\mu_{2}\left(b\beta_{1}+\alpha \mu_{1}\right)\right],\\ P_{1}=b^{2}\beta_{1}\beta_{2}\Lambda_{1}\mu_{1}-\sigma b\beta_{1}\Lambda_{1}b^{2}\beta_{1}\beta_{2}\frac{\Lambda_{2}}{\mu_{2}}+\sigma b\beta_{1}\mu_{1}^{2}\mu_{2}+\mu_{1}\mu_{2}\left(\mu_{1}+\gamma \right)\left(b\beta_{1}+\alpha \mu_{1}\right),\\ P_{0}=\mu_{1}^{2}\mu_{2}\left(\mu_{1}+\gamma \right)\left(1-R_{0}\right),\\ R_{0}=\frac{b^{2}\beta_{1}\beta_{2}\Lambda_{1}\Lambda_{2}}{\mu_{1}\mu_{2}^{2}\left(\mu_{1}+\gamma \right)}, \end{array}$$

where $V_{1}=\left(-P_{1}+\sqrt{P_{1}^{2}-4P_{0}P_{2}}\right)/2P_{2}$ and $V_{2}=\left(-P_{1}-\sqrt{P_{1}^{2}-4P_{0}P_{2}}\right)/2P_{2}$ are the two roots of (10) since *σ* ∈ [0,1], and we have

(12) $$\begin{array}{c} P_{1}\geq b^{2}\beta_{1}\beta_{2}\Lambda_{1}\mu_{1}+\sigma b\beta_{1}\mu_{1}^{2}\mu_{2}+\mu_{1}\mu_{2}\left(\mu_{1}+\gamma \right)\left(b\beta_{1}+\alpha \mu_{1}\right)-b\beta_{1}\Lambda_{1}b^{2}\beta_{1}\beta_{2}\frac{\Lambda_{2}}{\mu_{2}}=b^{2}\beta_{1}\beta_{2}\Lambda_{1}\mu_{1}+\sigma b\beta_{1}\alpha \mu_{1}^{2}\mu_{2}+b\beta_{1}\mu_{1}\mu_{2}\left(\mu_{1}+\gamma \right)\left(1-R_{0}\right). \end{array}$$

Obviously, *P*_2_ > 0, *P*_1_ > 0 and *P*_0_ > 0 if *R*_0_ < 1, and *P*_2_ > 0 and *P*_0_ < 0 if *R*_0_ > 1. From the relationship between roots and coefficients, we know that *V*_1_ and *V*_2_ are both negative if *R*_0_ < 1 and *V*_1_ is positive if *R*_0_ > 1. According to the above discussion, we can obtain the following theorems.

**Theorem 1 .**

System (7) has the disease-free equilibrium *E*_0_ if *R*_0_ < 1. System (7) has the disease-free equilibrium *E*_0_ and an endemic equilibrium *E*_1_ if *R*_0_ > 1.

## 3. Stability of Equilibrium and Hopf Bifurcation

In this section, we study the stability of equilibrium and the existence of Hopf bifurcation of system (7).

The characteristic equation of the linear approximate equation of the system (7) at equilibrium *E*=(*S*, *I*, *V*) is

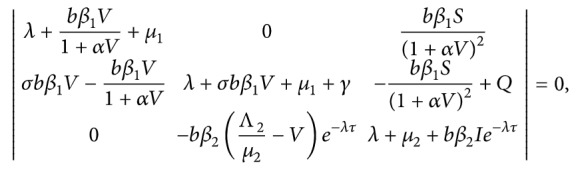
(13)where *Q*=−*σbβ*_1_((Λ_1_/*μ*_1_) − *S* − *I*).

### 3.1. The Local and Global Stability of the Disease-Free Equilibrium

At the disease-free equilibrium *E*_0_, equation (13) can be expressed as follows:

(14) $$\begin{array}{c} \left(\lambda +\mu_{1}\right)\left(\lambda^{2}+\left(\mu_{1}+\gamma +\mu_{2}\right)\lambda +\mu_{2}\left(\mu_{1}+\gamma \right)-b^{2}\beta_{1}\beta_{2}\frac{\Lambda_{1}\Lambda_{2}}{\mu_{1}\mu_{2}}e^{-\lambda \tau }\right)=0. \end{array}$$

Obviously, equation (14) has a negative real root *λ*_1_=−*μ*_1_. To discuss the rest of the characteristic roots of (14), we consider the following equation:

(15) $$\begin{array}{c} \lambda^{2}+\left(\mu_{1}+\gamma +\mu_{2}\right)\lambda +\mu_{2}\left(\mu_{1}+\gamma \right)-b^{2}\beta_{1}\beta_{2}\frac{\Lambda_{1}\Lambda_{2}}{\mu_{1}\mu_{2}}e^{-\lambda \tau }=0. \end{array}$$

When *τ*=0, equation (15) is equivalent to

(16) $$\begin{array}{c} \lambda^{2}+\left(\mu_{1}+\gamma +\mu_{2}\right)\lambda +\mu_{2}\left(\mu_{1}+\gamma \right)\left(1-R_{0}\right)=0. \end{array}$$

By the using Routh–Hurwitz criterion, (16) has two eigenvalues with negative real parts if *R*_0_ < 1.

When *τ* > 0, then the roots of (15) can enter the right-half plane in the complex plane by crossing the imaginary axis as the delay *τ* increases.

Let *λ*=*ωi*(*ω* > 0) be a purely imaginary root of equation (15), then separating the real and imaginary parts yields

(17) $$\begin{array}{c} \left\{\begin{array}{c} -\omega^{2}+\mu_{2}\left(\mu_{1}+\gamma \right)=b^{2}\beta_{1}\beta_{2}\frac{\Lambda_{1}\Lambda_{2}}{\mu_{1}\mu_{2}}\cos\;\omega \tau ,\\ \left(\mu_{2}+\mu_{1}+\gamma \right)\omega =-b^{2}\beta_{1}\beta_{2}\frac{\Lambda_{1}\Lambda_{2}}{\mu_{1}\mu_{2}}\sin\;\omega \tau . \end{array}\right. \end{array}$$

Squaring and taking the sum of (17) yields

(18) $$\begin{array}{c} \omega^{4}+\left(\mu_{2}^{2}+{\left(\mu_{1}+\gamma \right)}^{2}\right)\omega^{2}+\mu_{2}\left(\mu_{1}+\gamma \right)\left(1-R_{0}\right)\times \left(\mu_{2}\left(\mu_{1}+\gamma \right)+b^{2}\beta_{1}\beta_{2}\frac{\Lambda_{1}\Lambda_{2}}{\mu_{1}\mu_{2}}\right)=0. \end{array}$$

Equation (18) has no roots if *R*_0_ < 1. Therefore, we conclude that all eigenvalues of equation (14) have negative real parts.

If *R*_0_ > 1, let

(19) $$\begin{array}{c} f\left(\lambda \right)=\lambda^{2}+\left(\mu_{1}+\gamma +\mu_{2}\right)\lambda +\mu_{2}\left(\mu_{1}+\gamma \right)-b^{2}\beta_{1}\beta_{2}\frac{\Lambda_{1}\Lambda_{2}}{\mu_{1}\mu_{2}}e^{-\lambda \tau }, \end{array}$$

which implies that

(20) $$\begin{array}{c} f\left(0\right)=\mu_{2}\left(\mu_{1}+\gamma \right)\left(1-R_{0}\right)<0,\\ \underset{\lambda ⟶+\infty }{\lim}f\left(\lambda \right)=+\infty . \end{array}$$

By the continuity of *f*(*λ*) and zero point theorem, *f*(*λ*)=0 has at least one positive root. So, the disease-free equilibrium *E*_0_ is unstable. Based on the results, we can draw the conclusion.

**Theorem 2 .**

For any *τ*, the virus-free equilibrium *E*_0_ of the system (7) is locally asymptotically stable if *R*_0_ < 1, and it is unstable if *R*_0_ > 1.

In fact, using a similar approach to the literature [14], we can know that *E*_0_ is globally asymptotically stable if *R*_0_ < 1. A detailed proof is given below.

For a continuous and bounded function *f*(*t*), we define

(21) $$\begin{array}{c} f^{\infty }\;≜\;\underset{t⟶\infty }{\lim}\;\sup\;f\left(t\right),\\ f_{\infty }\;≜\;\underset{t⟶\infty }{\lim}\;\inf\;f\left(t\right). \end{array}$$

For system (7), any solution with the initial conditions is (*S*(*t*), *I*(*t*), *V*(*t*)), and we have

(22) $$\begin{array}{c} 0\leq S_{\infty }\leq S^{\infty }\leq \infty ,\\ 0\leq I_{\infty }\leq I^{\infty }\leq \infty ,\\ 0\leq V_{\infty }\leq V^{\infty }\leq \infty . \end{array}$$

By the fluctuation lemma [15], we know that there is a sequence {*t*_*n*_}; when *t*_*n*_⟶*∞*, we have *S*(*t*_*n*_)⟶*S* ^*∞*^ and *S*′(*t*_*n*_)⟶0(*n*⟶*∞*). Substituting *t*_*n*_ into the first equation of (7) yields

(23) $$\begin{array}{c} S^{\prime }\left(t_{n}\right)=\Lambda_{1}-\frac{b\beta_{1}S\left(t_{n}\right)V\left(t_{n}\right)}{1+\alpha V\left(t_{n}\right)}-\mu_{1}S\left(t_{n}\right)\leq \Lambda_{1}-\mu_{1}S\left(t_{n}\right). \end{array}$$

Let us take the limits on both sides:

(24) $$\begin{array}{c} \underset{n⟶\infty }{\lim}S^{\prime }\left(t_{n}\right)\leq \Lambda_{1}-\mu_{1}\underset{n⟶\infty }{\lim}S\left(t_{n}\right),\;\text{i}.\text{e}.,\;\;\mu_{1}S^{\infty }\leq \Lambda_{1}. \end{array}$$

Similarly,

(25) $$\begin{array}{c} \left(\mu_{1}+\gamma \right)I^{\infty }\leq \left(1-\sigma \right)b\beta_{1}S^{\infty }V^{\infty }+\sigma b\beta_{1}\frac{\Lambda_{1}}{\mu_{1}}V^{\infty },\\ \mu_{2}V^{\infty }\leq b\beta_{2}\frac{\Lambda_{2}}{\mu_{2}}I^{\infty }. \end{array}$$

Combining (24) and (25), we know that

(26) $$\begin{array}{c} \mu_{2}V^{\infty }\leq b^{2}\beta_{1}\beta_{2}\frac{\Lambda_{1}\Lambda_{2}}{\mu_{1}\mu_{2}\left(\mu_{1}+\gamma \right)}V^{\infty }. \end{array}$$

Since *V* ^*∞*^ is the supremum of the function *V*(*t*), *V* ^*∞*^ ≥ 0. If *V* ^*∞*^ > 0, by using (26), *μ*_2_ ≤ *b* ^2^ *β*_1_ *β*_2_(Λ_1_Λ_2_/*μ*_1_ *μ*_2_(*μ*_1_+*γ*)), which contradicts *R*_0_ < 1. That is to say, *V* ^*∞*^=0, which implies that $\underset{t⟶\infty }{\lim}V\left(t\right)=0$. In the same way by using (25), we have $\underset{t⟶\infty }{\lim}I\left(t\right)=0$. According to the limit theorem [13], we have $\underset{t⟶\infty }{\lim}S\left(t\right)=\Lambda_{1}/\mu_{1}$. Combined with the local asymptotic stability of *E*_0_, we can get the following theorem:

**Theorem 3 .**

For any *τ*, the virus-free equilibrium *E*_0_ of system (7) is globally asymptotically stable if *R*_0_ < 1.

### 3.2. The Local Stability of the Endemic Equilibrium

From (13), the characteristic equation of linear approximate equation of the system (7) at the endemic equilibrium *E*_1_ is

(27) $$\begin{array}{c} \lambda^{3}+A_{2}\lambda^{2}+A_{1}\lambda +A_{0}+\left(B_{2}\lambda^{2}+B_{1}\lambda +B_{0}\right)e^{-\lambda \tau }=0, \end{array}$$

where

(28) $$\begin{array}{c} A_{2}=\frac{b\beta_{1}V_{1}}{1+\alpha V_{1}}+2\mu_{1}+\sigma b\beta_{1}V_{1}+\gamma +\mu_{2},\\ A_{1}=\left(\frac{b\beta_{1}V_{1}}{1+\alpha V_{1}}+\mu_{1}\right)\left(\sigma b\beta_{1}V_{1}+\mu_{1}+\gamma +\mu_{2}\right)+\left(\sigma b\beta_{1}V_{1}+\mu_{1}+\gamma \right)\mu_{2},\\ A_{0}=\left(\frac{b\beta_{1}V_{1}}{1+\alpha V_{1}}+\mu_{1}\right)\left(\sigma b\beta_{1}V_{1}+\mu_{1}+\gamma \right)\mu_{2},\\ B_{2}=b\beta_{2}I_{1},\\ B_{1}=b\beta_{2}I_{1}\left(\frac{b\beta_{1}V_{1}}{1+\alpha V_{1}}+\sigma b\beta_{1}V_{1}+2\mu_{1}+\gamma \right)-\frac{b\beta_{1}S_{1}}{1+\alpha V_{1}}\left(\frac{1}{1+\alpha V_{1}}-1\right)\mu_{2}\frac{V_{1}}{I_{1}}-\mu_{2}\left(\mu_{1}+\gamma \right),\\ B_{0}=b\beta_{2}I_{1}\left(\frac{b\beta_{1}V_{1}}{1+\alpha V_{1}}+\mu_{1}\right)\left(\sigma b\beta_{1}V_{1}+\mu_{1}+\gamma \right)-\frac{b\beta_{1}S_{1}}{1+\alpha V_{1}}\left(\frac{b\beta_{1}V_{1}}{1+\alpha V_{1}}+\mu_{1}\right)\left(\frac{1}{1+\alpha V_{1}}-1\right)\times \mu_{2}\frac{V_{1}}{I_{1}}-\left(\frac{b\beta_{1}V_{1}}{1+\alpha V_{1}}+\mu_{1}\right)\mu_{2}\left(\mu_{1}+\gamma \right)+\left(\frac{b\beta_{1}V_{1}}{1+\alpha V_{1}}-\sigma b\beta_{1}V_{1}\right)\frac{b\beta_{1}S_{1}}{{\left(1+\alpha V_{1}\right)}^{2}}\mu_{2}\frac{V_{1}}{I_{1}}. \end{array}$$

When *τ*=0, equation (27) is equivalent to

(29) $$\begin{array}{c} \lambda^{3}+H_{2}\lambda^{2}+H_{1}\lambda +H_{0}=0, \end{array}$$

where

(30) $$\begin{array}{c} H_{2}=A_{2}+B_{2}=\frac{b\beta_{1}V_{1}}{1+\alpha V_{1}}+b\beta_{2}I_{1}+2\mu_{1}+\mu_{2}+\sigma b\beta_{1}V_{1}+\gamma >0,\\ H_{1}=A_{1}+B_{1}=\left(\frac{b\beta_{1}V_{1}}{1+\alpha V_{1}}+\mu_{1}\right)\left(\sigma b\beta_{1}V_{1}+\mu_{1}+\gamma +\mu_{2}\right)+\sigma b\beta_{1}V_{1}\mu_{2}+b\beta_{2}I_{1}\left(\frac{b\beta_{1}V_{1}}{1+\alpha V_{1}}+\sigma b\beta_{1}V_{1}+2\mu_{1}+\gamma \right)+\frac{b\beta_{1}S_{1}}{1+\alpha V_{1}}\left(1-\frac{1}{1+\alpha V_{1}}\right)\mu_{2}\frac{V_{1}}{I_{1}}>0,\\ H_{0}=A_{0}+B_{0}=\left(\frac{b\beta_{1}V_{1}}{1+\alpha V_{1}}+\mu_{1}\right)\left[\left(\sigma b\beta_{1}V_{1}+\mu_{1}+\gamma \right)b\beta_{2}I_{1}+\sigma b\beta_{1}V_{1}\mu_{2}\right]+\left(1-\sigma \right)b\beta_{1}V_{1}\frac{b\beta_{1}S_{1}}{{\left(1+\alpha V_{1}\right)}^{2}}\frac{\mu_{2}V_{1}}{I_{1}}+\mu_{1}\frac{b\beta_{1}S_{1}}{1+\alpha V_{1}}\left(1-\frac{1}{1+\alpha V_{1}}\right)\frac{\mu_{2}V_{1}}{I_{1}}>0. \end{array}$$

Notice that

(31) $$\begin{array}{c} H_{2}H_{1}-H_{0}=\left(\frac{b\beta_{1}V_{1}}{1+\alpha V_{1}}+b\beta_{2}I_{1}+2\mu_{1}+\mu_{2}+\sigma b\beta_{1}V_{1}+\gamma \right)\left[\left(\frac{b\beta_{1}V_{1}}{1+\alpha V_{1}}+\mu_{1}\right)\times \left(\sigma b\beta_{1}V_{1}+\mu_{1}+\gamma +\mu_{2}\right)+\sigma b\beta_{1}V_{1}\mu_{2}+b\beta_{2}I_{1}\left(\frac{b\beta_{1}V_{1}}{1+\alpha V_{1}}+\sigma b\beta_{1}V_{1}+2\mu_{1}+\gamma \right)+\frac{b\beta_{1}S_{1}}{1+\alpha V_{1}}\left(1-\frac{1}{1+\alpha V_{1}}\right)\mu_{2}\frac{V_{1}}{I_{1}}\right]-\left(\frac{b\beta_{1}V_{1}}{1+\alpha V_{1}}+\mu_{1}\right)\left[\left(\sigma b\beta_{1}V_{1}+\mu_{1}+\gamma \right)b\beta_{2}I_{1}+\sigma b\beta_{1}V_{1}\mu_{2}\right]-\left(1-\sigma \right)b\beta_{1}V_{1}\frac{b\beta_{1}S_{1}}{{\left(1+\alpha V_{1}\right)}^{2}}\frac{\mu_{2}V_{1}}{I_{1}}-\mu_{1}\frac{b\beta_{1}S_{1}}{1+\alpha V_{1}}\left(1-\frac{1}{1+\alpha V_{1}}\right)\frac{\mu_{2}V_{1}}{I_{1}}\geq \left(\frac{b\beta_{1}V_{1}}{1+\alpha V_{1}}+\mu_{1}+\mu_{2}\right)\left[\frac{b\beta_{1}V_{1}}{1+\alpha V_{1}}\left(\gamma +\mu_{1}\right)+\sigma b\beta_{1}V_{1}\mu_{2}+b\beta_{2}I_{1}\times \left(\sigma b\beta_{1}V_{1}+\mu_{1}+\gamma \right)\right]+\frac{b\beta_{1}S_{1}}{1+\alpha V_{1}}\left(1-\frac{1}{1+\alpha V_{1}}\right)\mu_{2}\frac{V_{1}}{I_{1}}\left(\frac{b\beta_{1}V_{1}}{1+\alpha V_{1}}+\mu_{1}\right)-\left(\frac{b\beta_{1}V_{1}}{1+\alpha V_{1}}+\mu_{1}\right)\left[\left(\sigma b\beta_{1}V_{1}+\mu_{1}+\gamma \right)b\beta_{2}I_{1}+\sigma b\beta_{1}V_{1}\mu_{2}\right]-\left(1-\sigma \right)b\beta_{1}V_{1}\frac{b\beta_{1}S_{1}}{{\left(1+\alpha V_{1}\right)}^{2}}\frac{\mu_{2}V_{1}}{I_{1}}-\mu_{1}\frac{b\beta_{1}S_{1}}{1+\alpha V_{1}}\left(1-\frac{1}{1+\alpha V_{1}}\right)\frac{\mu_{2}V_{1}}{I_{1}}\geq \frac{b\beta_{1}V_{1}}{1+\alpha V_{1}}\mu_{2}\left(\gamma +\mu_{1}\right)-\frac{b\beta_{1}S_{1}}{{\left(1+\alpha V_{1}\right)}^{2}}\frac{\mu_{2}V_{1}}{I_{1}}\frac{b\beta_{1}V_{1}}{1+\alpha V_{1}}+\sigma b\beta_{1}V_{1}\frac{b\beta_{1}S_{1}}{{\left(1+\alpha V_{1}\right)}^{2}}\frac{\mu_{2}V_{1}}{I_{1}}, \end{array}$$

(32) $$\begin{array}{c} \frac{V_{1}}{I_{1}}=\frac{\mu_{1}+\gamma }{\left(b\beta_{1}S_{1}/1+\alpha V_{1}\right)+\sigma b\beta_{1}\left(\left(\Lambda_{1}/\mu_{1}\right)-S_{1}-I_{1}\right)}\leq \frac{\mu_{1}+\gamma }{\left(b\beta_{1}S_{1}/\left(1+\alpha V_{1}\right)\right)}=\frac{\left(\mu_{1}+\gamma \right)\left(1+\alpha V_{1}\right)}{b\beta_{1}S_{1}}. \end{array}$$

It follows that

(33) $$\begin{array}{c} H_{2}H_{1}-H_{0}\geq \frac{b\beta_{1}V_{1}}{1+\alpha V_{1}}\mu_{2}\left(\gamma +\mu_{1}\right)-\frac{b\beta_{1}S_{1}}{{\left(1+\alpha V_{1}\right)}^{2}}\frac{b\beta_{1}V_{1}}{1+\alpha V_{1}}\mu_{2}\frac{\left(\mu_{1}+\gamma \right)\left(1+\alpha V_{1}\right)}{b\beta_{1}S_{1}}=\frac{b\beta_{1}V_{1}}{1+\alpha V_{1}}\mu_{2}\left(\gamma +\mu_{1}\right)-\frac{b\beta_{1}V_{1}}{{\left(1+\alpha V_{1}\right)}^{2}}\left(\gamma +\mu_{1}\right)\mu_{2}=\frac{b\beta_{1}V_{1}}{1+\alpha V_{1}}\mu_{2}\left(\gamma +\mu_{1}\right)\left(1-\frac{1}{1+\alpha V_{1}}\right)>0. \end{array}$$

By using the Routh–Hurwitz criterion, equation (29) only has eigenvalues with negative real parts if *R*_0_ > 1. We can obtain the following theorem:

**Theorem 4 .**

For *τ*=0, the endemic equilibrium *E*_1_ of system (7) is locally asymptotically stable if *R*_0_ > 1.

### 3.3. Hopf Bifurcation

In this subsection, we devote to investigating the stability of the endemic equilibrium and the existence of Hopf bifurcation.

Let *λ*=*ωi*(*ω* > 0) be the root of equation (27), substituting it into equation (27) and separating the real and imaginary parts; we can obtain the following equation:

(34) $$\begin{array}{c} \left\{\begin{array}{c} A_{2}\omega^{2}-A_{0}=\left(B_{0}-B_{2}\omega^{2}\right)\cos\;\omega \tau +B_{1}\omega \;\sin\;\omega \tau ,\\ \omega^{3}-A_{1}\omega =B_{1}\omega \;\cos\;\omega \tau -\left(B_{0}-B_{2}\omega^{2}\right)\sin\;\omega \tau . \end{array}\right. \end{array}$$

Squaring and taking the sum of (34) yields

(35) $$\begin{array}{c} \omega^{6}+p_{2}\omega^{4}+p_{1}\omega^{2}+p_{0}=0, \end{array}$$

where

(36) $$\begin{array}{c} p_{2}=A_{2}^{2}-2A_{1}-B_{2}^{2},\\ p_{1}=A_{1}^{2}-2A_{0}A_{2}+2B_{0}B_{2}-B_{1}^{2},\\ p_{0}=A_{0}^{2}-B_{0}^{2}. \end{array}$$

Let *x*=*ω* ^2^, then equation (35) is equivalent to

(37) $$\begin{array}{c} f\left(x\right)\;≜\;x^{3}+p_{2}x^{2}+p_{1}x+p_{0}=0, \end{array}$$

then *f*′(*x*)=3*x* ^2^+2*p*_2_ *x*+*p*_1_. The two roots of equation 3*x* ^2^+2*p*_2_ *x*+*p*_1_=0 are

(38) $$\begin{array}{c} x^{*}=\frac{-p_{2}+\sqrt{p_{2}^{2}-3p_{1}}}{3},\\ x^{**}=\frac{-p_{2}-\sqrt{p_{2}^{2}-3p_{1}}}{3}. \end{array}$$

According to [16], the condition that equation (37) has positive roots is as follows:

**Lemma 1 .**

For equation (37),

- If *p*_0_ < 0, then equation (37) has at least one positive root
- If *p*_0_ ≥ 0 and *p*_2_^2^ ≤ 3*p*_1_, then equation (37) has no positive root
- If *p*_0_ ≥ 0 and *p*_2_^2^ > 3*p*_1_, then equtaion (37) has positive roots if and only if *x* ^*∗*^ > 0 and *f*(*x* ^*∗*^) ≤ 0

Based on Lemma 3.4, we concluded that if (ii) is set up, then the stability of *E*_1_ will not change when *τ* increases. If equation (37) has a positive root, then the stability of *E*_1_ may change with the change in *τ*.

Suppose that equation (37) has three positive roots, written as *x*_1_, *x*_2_, and *x*_3_. Then, equation (35) has positive roots $\omega_{k}=\sqrt{x_{k}}$(*k*=1,2,3). By using (34),

(39) $$\begin{array}{c} \cos\;\omega \tau =\frac{\left(B_{0}-B_{2}\omega^{2}\right)\left(A_{2}\omega^{2}-A_{0}\right)+B_{1}\omega \left(\omega^{3}-A_{1}\omega \right)}{{\left(B_{0}-B_{2}\omega^{2}\right)}^{2}+{\left(B_{1}\omega \right)}^{2}}. \end{array}$$

Define

(40) $$\begin{array}{c} \tau_{k}^{\left(j\right)}=\frac{1}{\omega_{k}}\left\{\arccos\frac{\left(B_{0}-B_{2}\omega_{k}^{2}\right)\left(A_{2}\omega_{k}^{2}-A_{0}\right)+B_{1}\omega_{k}\left(\omega_{k}^{3}-A_{1}\omega_{k}\right)}{{\left(B_{0}-B_{2}\omega_{k}^{2}\right)}^{2}+{\left(B_{1}\omega_{k}\right)}^{2}}+2j\pi \right\}, \end{array}$$

where *k*=1,2,3, *j*=0,1,…. Obviously, ±*ω*_*k*_ *i* is a pair of pure virtual root of equation (27).

Let

(41) $$\begin{array}{c} \tau^{*}=\tau_{k_{0}}^{\left(0\right)}=\underset{k\in \left\{1,2,3\right\}}{\min}\left\{\tau_{k}^{\left(0\right)}\right\},\\ \omega^{*}=\omega_{k_{0}}. \end{array}$$

It follows that *λ*(*τ*)=*ξ*_0_(*τ*)+*iω*(*τ*) is the root of equation (35) satisfying *ξ*_0_(*τ* ^*∗*^)=0 and *ω*(*τ* ^*∗*^)=*ω* ^*∗*^.

Next, we verify the transversal condition. Differentiating the two sides of equation (35) with respect to *τ*, we have

(42) $$\begin{array}{c} {\left(\frac{d\lambda }{d\tau }\right)}^{-1}=-\frac{3\lambda^{2}+2A_{2}\lambda +A_{1}}{\lambda \left(\lambda^{3}+A_{2}\lambda^{2}+A_{1}\lambda +A_{0}\right)}+\frac{2\lambda B_{2}+B_{0}}{\lambda \left(B_{2}\lambda^{2}+B_{1}\lambda +B_{0}\right)}-\frac{\tau }{\lambda }, \end{array}$$

then

(43) Redλdτ−1τ=τ∗=3ω∗4+2A22−2A1−B22ω∗2+A12−2A0A2+2B0B2−B12B1ω∗2+B0−B2ω∗22=f′ω∗2B1ω∗2+B0−B2ω∗22.

Thus,

(44) signdReλdττ=τ∗=signRedλdτ−1τ=τ∗=signf′ω∗2.

If *f*′(*ω* ^*∗*2^) > 0, the transversal condition is satisfied. Therefore, according to the above discussion and the Hopf bifurcation theorem of the differential equations [12], we can get the following result:

**Theorem 5 .**

- If *p*_0_ ≥ 0 and Δ  =  *p*_2_^2^ − 3*p*_1_ ≤ 0, then the endemic equilibrium *E*_1_ is locally asymptotically stable for all *τ* > 0.
- If *p*_0_ < 0 or *p*_0_ ≥ 0, Δ > 0, *x* ^*∗*^ > 0, and *f*(*x* ^*∗*^) ≤ 0, then the endemic equilibrium *E*_1_ is locally asymptotically stable for 0 < *τ* < *τ* ^*∗*^, and if *f*′(*ω* ^*∗*^2^^) ≠ 0, the system (7) undergoes a Hopf bifurcation at *E*_1_ when *τ*=*τ* ^*∗*^, where

(45) $$\begin{array}{c} \tau^{*}=\frac{1}{\omega^{*}}\left\{\arccos\frac{\left(B_{0}-B_{2}\omega^{{*}^{2}}\right)\left(A_{2}\omega^{{*}^{2}}-A_{0}\right)+B_{1}\omega^{*}\left(\omega^{{*}^{3}}-A_{1}\omega^{*}\right)}{{\left(B_{0}-B_{2}\omega^{{*}^{2}}\right)}^{2}+{\left(B_{1}\omega^{*}\right)}^{2}}\right\}. \end{array}$$

## 4. Estimation of the Length of Delay to Preserve Stability

In this section, we use a Nyquist criterion [17] to calculate the length of delay to preserve stability.

Consider the system (7) and the space of the real continuous functions that is defined in [−*τ*, +*∞*] and satisfied the initial conditions (2) in the interval [−*τ*, 0]. Define

(46) $$\begin{array}{c} S\left(t\right)=S_{1}+X\left(t\right),\\ I\left(t\right)=I_{1}+Y\left(t\right),\\ V\left(t\right)=V_{1}+Z\left(t\right). \end{array}$$

Linearization system (7) at the endemic equilibrium *E*_1_ is expressed as follows:

(47) $$\begin{array}{c} \left\{\begin{array}{c} \frac{dX\left(t\right)}{dt}=-\left(\frac{b\beta_{1}V_{1}}{1+\alpha V_{1}}+\mu_{1}\right)X\left(t\right)-\frac{b\beta_{1}S_{1}}{{\left(1+\alpha V_{1}\right)}^{2}}Z\left(t\right),\\ \frac{dY\left(t\right)}{dt}=\left(\frac{b\beta_{1}V_{1}}{1+\alpha V_{1}}-\sigma b\beta_{1}V_{1}\right)X\left(t\right)-\left(\sigma b\beta_{1}V_{1}+\mu_{1}+\gamma \right)Y\left(t\right)+\left[\frac{b\beta_{1}S_{1}}{{\left(1+\alpha V_{1}\right)}^{2}}+\sigma b\beta_{1}\left(\frac{\Lambda_{1}}{\mu_{1}}-S_{1}-I_{1}\right)\right]Z\left(t\right),\\ \frac{dZ\left(t\right)}{dt}=-\mu_{2}Z\left(t\right)+b\beta_{2}\left(\frac{\Lambda_{2}}{\mu_{2}}-V_{1}\right)Y\left(t-\tau \right)-b\beta_{2}I_{1}Z\left(t-\tau \right). \end{array}\right. \end{array}$$

By taking the Laplace transformation for (47), we can obtain

(48) $$\begin{array}{c} \left\{\begin{array}{c} SL\left[X\right]-X\left(0\right)=-\left(\frac{b\beta_{1}V_{1}}{1+\alpha V_{1}}+\mu_{1}\right)L\left[X\right]-\frac{b\beta_{1}S_{1}}{{\left(1+\alpha V_{1}\right)}^{2}}L\left[Z\right],\\ SL\left[Y\right]-Y\left(0\right)=\left(\frac{b\beta_{1}V_{1}}{1+\alpha V_{1}}-\sigma b\beta_{1}V_{1}\right)L\left[X\right]-\left(\sigma b\beta_{1}V_{1}+\mu_{1}+\gamma \right)L\left[Y\right]+\left[\frac{b\beta_{1}S_{1}}{{\left(1+\alpha V_{1}\right)}^{2}}+\sigma b\beta_{1}\left(\frac{\Lambda_{1}}{\mu_{1}}-S_{1}-I_{1}\right)\right]L\left[Z\right],\\ SL\left[Z\right]-Z\left(0\right)=-\mu_{2}L\left[Z\right]+b\beta_{2}\left(\frac{\Lambda_{2}}{\mu_{2}}-V_{1}\right)L\left[Y_{\tau }\right]-b\beta_{2}I_{1}L\left[Z_{\tau }\right], \end{array}\right. \end{array}$$

where

(49) $$\begin{array}{c} L\left[Y_{\tau }\right]=\int_{0}^{\infty }e^{-st}Y\left(t-\tau \right)dt=\int_{0}^{\tau }e^{-st}Y\left(t-\tau \right)dt+\int_{\tau }^{\infty }e^{-st}Y\left(t-\tau \right)dt. \end{array}$$

Let *t*=*t*_1_+*τ*, then

(50) $$\begin{array}{c} L\left[Y_{\tau }\right]=\int_{-\tau }^{0}e^{-s\left(t_{1}+\tau \right)}Y\left(t_{1}\right)dt_{1}+\int_{0}^{\infty }e^{-s\left(t_{1}+\tau \right)}Y\left(t_{1}\right)dt_{1}=M_{1}e^{-s\tau }+e^{-s\tau }L\left[Y\right], \end{array}$$

where *M*_1_=∫_−*τ*_^0^ *e* ^−*st*^ *Y*(*t*)*dt*.

Similarly,

(51) $$\begin{array}{c} L\left[Y_{\tau }\right]=\int_{-\tau }^{0}e^{-s\left(t_{1}+\tau \right)}Y\left(t_{1}\right)dt_{1}+\int_{0}^{\infty }e^{-s\left(t_{1}+\tau \right)}Y\left(t_{1}\right)dt_{1}=M_{1}e^{-s\tau }+e^{-s\tau }L\left[Y\right], \end{array}$$

where *M*_2_=∫_−*τ*_^0^ *e* ^−*st*^ *Z*(*t*)*dt*.

Thus, (48) can be written as

(52) $$\begin{array}{c} \left(A-SI\right)\left(\begin{array}{c} L\left[X\right]\\ L\left[Y\right]\\ L\left[Z\right] \end{array}\right)=B, \end{array}$$

where

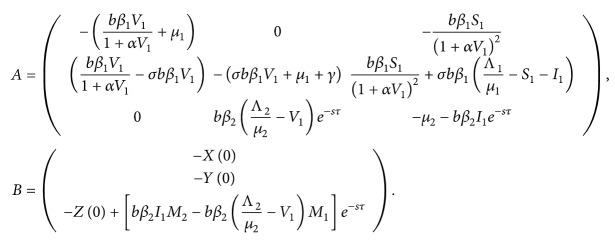
(53)


The inverse Laplace transformation of *L*[*X*(*t*)], *L*[*Y*(*t*)], and *L*[*Z*(*t*)] will have terms which exponentially increase with time if *L*[*X*(*t*)], *L*[*Y*(*t*)], and *L*[*Z*(*t*)] have poles with positive real parts. Thus, *E*_1_ is locally asymptotically stable if and only if all the poles of *L*[*X*(*t*)], *L*[*Y*(*t*)], and *L*[*Z*(*t*)] have negative real parts.

By the method of [17] and the Nyquist criterion, the local asymptotic stability of *E*_1_ needs to satisfy the following two conditions:

(54) Re Fiμ0=0,

(55) Im Fiμ0>0,

where

(56) $$\begin{array}{c} F\left(s\right)=s^{3}+A_{2}s^{2}+A_{1}s+A_{0}+\left(B_{2}s^{2}+B_{1}s+B_{0}\right)e^{-s\tau }, \end{array}$$

where *μ*_0_ is the smallest positive root of (54). Thus (54) and (55) can be written as

(57) $$\begin{array}{c} A_{2}\mu_{0}^{2}-A_{0}=\left(B_{0}-B_{2}\mu_{0}^{2}\right)\cos\;\mu_{0}\tau +B_{1}\mu_{0}\sin\;\mu_{0}\tau ,\\ -\mu_{0}^{3}+A_{1}\mu_{0}>\left(B_{0}-B_{2}\mu_{0}^{2}\right)\sin\;\mu_{0}\tau -B_{1}\mu_{0}\cos\;\mu_{0}\tau . \end{array}$$

In order to estimate the length of delay to preserve stability, under the premise of ensuring stability, the following conditions need to be satisfied:

(58) $$\begin{array}{c} A_{2}\mu^{2}-A_{0}=\left(B_{0}-B_{2}\mu^{2}\right)\cos\;\mu \tau +B_{1}\mu \;\sin\;\mu \tau , \end{array}$$

(59) $$\begin{array}{c} -\mu^{3}+A_{1}\mu >\left(B_{0}-B_{2}\mu^{2}\right)\sin\;\mu \tau -B_{1}\mu \;\cos\;\mu \tau . \end{array}$$

If (58) and (59) are satisfied simultaneously, they are sufficient conditions to guarantee stability. Our aim is to find an upper bound *μ*_+_ to *μ*_0_ independent of *τ* and then to estimate *τ* so that (59) holds true for all values of 0 ≤ *μ* ≤ *μ*_+_ and in particular at *μ*=*μ*_0_.

Since |cos *μτ*| ≤ 1 and |sin *μτ*| ≤ 1, from equation (58), we have

(60) $$\begin{array}{c} A_{2}\mu^{2}\leq \left|B_{0}\right|+B_{2}\mu^{2}+\left|B_{1}\right|\mu +A_{0}. \end{array}$$

Let

(61) $$\begin{array}{c} \mu_{+}=\frac{\left|B_{1}\right|+\sqrt{B_{1}^{2}+4\left(A_{2}-B_{2}\right)\left(A_{0}+\left|B_{0}\right|\right)}}{2\left(A_{2}-B_{2}\right)}, \end{array}$$

obviously *μ*_+_ meets (60) and *μ*_+_ ≥ *μ*_0_.

From equation (59), we obtain

(62) $$\begin{array}{c} \mu^{2}<B_{1}\cos\;\mu \tau +\left(B_{2}\mu -\frac{B_{0}}{\mu }\right)\sin\;\mu \tau +A_{1}. \end{array}$$

Since *E*_1_ is locally asymptotically stable for *τ*  =  0, the inequality (62) will continue to hold for sufficiently small *τ* and *μ*=*μ*_0_.

On the basis of (58) and (62), we have

(63) $$\begin{array}{c} \left(B_{1}\mu -A_{2}B_{2}\mu +\frac{A_{2}B_{0}}{\mu }\right)\sin\;\mu \tau +\left(B_{2}\mu^{2}+A_{2}B_{1}-B_{0}\right)\left(1-\cos\;\mu \tau \right)<B_{2}\mu^{2}+A_{1}A_{2}+A_{2}B_{1}-A_{0}-B_{0}. \end{array}$$

Note the left-hand side of (63) is Φ(*τ*, *μ*) and the right-hand side is *ρ*. By using inequality sin *μτ* ≤ *μτ* and 1 − cos *μτ*=2 sin^2^(*μτ*/2) ≤ (*μ* ^2^ *τ* ^2^/2), we can obtain

(64) $$\begin{array}{c} \Phi \left(\tau ,\mu \right)\leq \left|\Phi \left(\tau ,\mu \right)\right|\leq \left(\left|B_{1}-A_{2}B_{2}\right|\mu +\left|\frac{A_{2}B_{0}}{\mu }\right|\right)\mu \tau \;\;+\left|B_{2}\mu^{2}+A_{2}B_{1}-B_{0}\right|\frac{\mu^{2}\tau^{2}}{2}=\left(\left|B_{1}-A_{2}B_{2}\right|\mu^{2}+\left|A_{2}B_{0}\right|\right)\tau +\left|B_{2}\mu^{2}+A_{2}B_{1}-B_{0}\right|\frac{\mu^{2}\tau^{2}}{2}. \end{array}$$

Note the right-hand side of (64) is *φ*(*τ*, *μ*). Clearly, Φ(*τ*, *μ*) ≤ *φ*(*τ*, *μ*) ≤ *φ*(*τ*, *μ*_+_) when *μ* ∈ [0, *μ*_+_]. Thus, if *φ*(*τ*, *μ*_+_) ≤ *ρ* ≤ *K*_3_, we have Φ(*τ*, *μ*_+_) ≤ *ρ* ≤ *K*_3_. Let *τ* ^*∗*^ be the positive root of *φ*(*τ*, *μ*_+_)=*K*_3_, that is,

(65) $$\begin{array}{c} \tau^{*}=\frac{1}{2K_{1}}\left(-K_{2}+\sqrt{K_{2}^{2}+4K_{1}K_{3}}\right), \end{array}$$

where

(66) $$\begin{array}{c} K_{1}=\frac{\left|B_{2}\mu_{+}^{2}+A_{2}B_{1}-B_{0}\right|\mu_{+}^{2}}{2},\\ K_{2}=\left|B_{1}-A_{2}B_{2}\right|\mu_{+}^{2}+\left|A_{2}B_{0}\right|,\\ K_{3}=B_{2}\mu_{+}^{2}+A_{1}A_{2}+A_{2}B_{1}-A_{0}-B_{0}. \end{array}$$

Summarizing the above discussions, we have the following theorem.

**Theorem 6 .**

If 0 < *τ* < *τ* ^*∗*^, then the Nyquist criterion holds true and *τ* ^*∗*^ estimates the maximum length of the delay preserving the stability, where *τ* ^*∗*^ satisfies (65).

## 5. Direction and Stability of the Hopf Bifurcation

We have obtained the conditions under which the Hopf bifurcation occurs at *E*_1_ of the system (7). This section will use the normal form theory and the center manifold theory to give the direction of the Hopf bifurcation and the stability of the bifurcating periodic solutions of system (7). We suppose that system (7) undergoes Hopf bifurcation at *E*_1_ for $\tau =\;\;\tilde{\tau }\left(\tilde{\tau }=\tau_{k}^{\left(j\right)}\right)$. Let ±*iω* be a pair of conjugate pure virtual roots at *E*_1_ when $\tau =\tilde{\tau }$.

Define

(67) $$\begin{array}{c} x_{1}\left(t\right)=S\left(\tau t\right)-S_{1},\\ x_{2}\left(t\right)=I\left(\tau t\right)-I_{1},\\ x_{3}\left(t\right)=V\left(\tau t\right)-V_{1},\\ \tau =\tilde{\tau }+\mu . \end{array}$$

Thus, system (7) is equivalent to the following functional differential equation in *C*=*C*([−*τ*, 0], *R* ^3^).

(68) $$\begin{array}{c} \frac{dx}{dt}=L_{\mu }\left(x_{t}\right)+f\left(\mu ,x_{t}\right), \end{array}$$

where *x*(*t*)=(*x*_1_(*t*), *x*_2_(*t*), *x*_3_(*t*))^T^  ∈*R* ^3^. And *L*_*μ*_ : *C*⟶*R* ^3^ and *f* : *R* × *C*⟶*R* ^3^ satisfy

(69) $$\begin{array}{c} L_{\mu }\left(\phi \right)=\left(\tilde{\tau }+\mu \right)B_{1}\phi \left(0\right)+\left(\tilde{\tau }+\mu \right)B_{2}\phi \left(-1\right), \end{array}$$

(70) $$\begin{array}{c} f\left(\mu ,\phi \right)=\left(\tilde{\tau }+\mu \right){\left(f_{1},f_{2},f_{3}\right)}^{\text{T}}, \end{array}$$

where

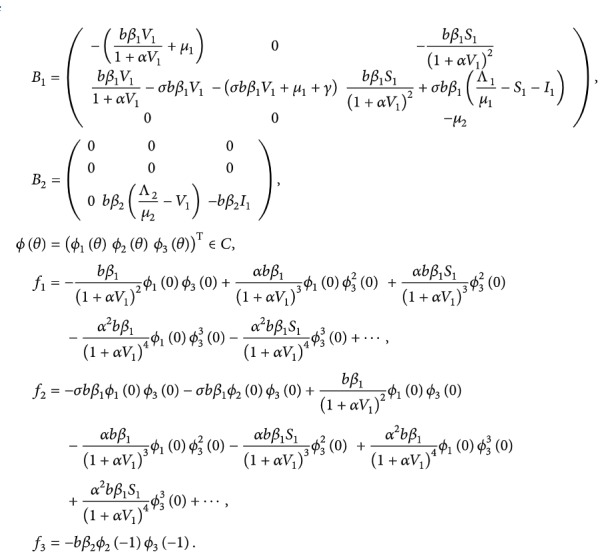
(71)


Applying the Riesz representation theorem, there exists a 3 × 3 matrix-valued function *η*(·, *μ*) : [−1,0]⟶*R* ^3×3^, such that *L*_*μ*_ *ϕ*=∫_−1_^0^ *dη*(*θ*, *μ*)*ϕ*(*θ*), *ϕ* ∈ *C*. We choose

(72) $$\begin{array}{c} \eta \left(\theta ,\mu \right)=\left(\tilde{\tau }+\mu \right)B_{1}\delta \left(\theta \right)+\left(\tilde{\tau }+\mu \right)B_{2}\delta \left(\theta +1\right), \end{array}$$

where *δ* is the Dirac delta function, meeting *δ*(*θ*)=0(*θ* ≠ 0) and ∫_−*∞*_^+*∞*^ *δ*(*θ*)*dθ*=1.

We define for *ϕ* ∈ *C*([−1,0], *R* ^3^),

(73) $$\begin{array}{c} A\left(\mu \right)\phi =\left\{\begin{array}{cc} \frac{d\phi \left(\theta \right)}{d\theta }, & \theta \in \left[-1,0\right),\\ \int_{-1}^{0}d\eta \left(s,\mu \right)\phi \left(s\right), & \theta =0, \end{array}\right.\\ R\left(\mu \right)\phi =\left\{\begin{array}{cc} 0, & \theta \in \left[-1,0\right),\\ f\left(\mu ,\phi \right), & \theta =0. \end{array}\right. \end{array}$$

Thus, (68) becomes

(74) $$\begin{array}{c} {\dot{x}}_{t}=A\left(\mu \right)x_{t}+R\left(\mu \right)x_{t}, \end{array}$$

where *x*_*t*_=*x*(*t*+*θ*), *θ* ∈ [−1,0].

In order to construct coordinates to describe the integral manifold near the origin, we need to define inner product and the adjoint operator *A* ^*∗*^=*A* ^*∗*^(0) of *A* as follows:

(75) $$\begin{array}{c} A^{*}\psi \left(s\right)=\left\{\begin{array}{cc} -\frac{d\psi \left(s\right)}{ds}, & s\in \left(0,1\right],\\ \int_{-1}^{0}d\eta^{\text{T}}\left(t,0\right)\psi \left(-t\right), & s=0, \end{array}\right.\\ \langle \psi ,\phi \rangle ={\bar{\psi }}^{\text{T}}\left(0\right)\phi \left(0\right)-\int_{\theta =-1}^{0}\int_{\xi =0}^{\theta }{\bar{\psi }}^{\text{T}}\left(\xi -\theta \right)d\eta \left(\theta \right)\phi \left(\xi \right)d\xi , \end{array}$$

where *ψ* ∈ *C*([0,1], *R* ^3^) and *η*(*θ*)=*η*(*θ*, 0). Form the discussion in Section 3, we know that $\pm i\omega \tilde{\tau }$ are eigenvalues of *A*(0). Thus they are also eigenvalues of *A* ^*∗*^. Define $q\left(\theta \right)={\left(1,q_{1},q_{2}\right)}^{\text{T}}e^{i\theta \omega \tilde{\tau }}$ and $q^{*}\left(s\right)=D{\left(1,q_{1}^{*},q_{2}^{*}\right)}^{\text{T}}e^{-is\omega \tilde{\tau }}$ to be the eigenvectors of *A*(0) and *A* ^*∗*^ corresponding to the eigenvalues $i\omega \tilde{\tau }$ and $-i\omega \tilde{\tau }$, then

(76) $$\begin{array}{c} A\left(0\right)q\left(\theta \right)=i\omega \tilde{\tau }q\left(\theta \right),\\ A^{*}q^{*}\left(s\right)=-i\omega \tilde{\tau }q^{*}\left(s\right). \end{array}$$

We can calculate that

(77) $$\begin{array}{c} q_{1}=\frac{I_{1}\left(\mu_{2}+b\beta_{2}I_{1}e^{-i\omega \tilde{\tau }}+\omega i\right)\left[b\beta_{1}V_{1}\left(1+\alpha V_{1}\right)+{\left(1+\alpha V_{1}\right)}^{2}\omega i\right]}{b\beta_{1}S_{1}\mu_{2}V_{1}e^{-i\omega \tilde{\tau }}},\\ q_{2}=\left(1+\alpha V_{1}\right)\frac{V_{1}}{S_{1}}+\frac{{\left(1+\alpha V_{1}\right)}^{2}\omega i}{b\beta_{1}S_{1}},\\ q_{1}^{*}=\frac{b\beta_{1}V_{1}+\left(\mu_{1}-\omega i\right)\left(1+\alpha V_{1}\right)}{b\beta_{1}V_{1}-\sigma b\beta_{1}V_{1}\left(1+\alpha V_{1}\right)},\\ q_{2}^{*}=\frac{I_{1}\left(\sigma b\beta_{1}V_{1}+\mu_{1}+\gamma -\omega i\right)\left[b\beta_{1}V_{1}+\left(\mu_{1}-\omega i\right)\left(1+\alpha V_{1}\right)\right]}{\mu_{2}V_{1}e^{i\omega \tilde{\tau }}\left[b\beta_{1}V_{1}-\sigma b\beta_{1}V_{1}\left(1+\alpha V_{1}\right)\right]}. \end{array}$$

According to (75), we know that

(78) $$\begin{array}{c} \langle q^{*}\left(s\right),q\left(\theta \right)\rangle =\bar{D}\left(1,{\bar{q}}_{1}^{*},{\bar{q}}_{2}^{*}\right){\left(1,q_{1},q_{2}\right)}^{\text{T}}-\int_{-1}^{0}\int_{\xi =0}^{\theta }\bar{D}\left(1,{\bar{q}}_{1}^{*},{\bar{q}}_{2}^{*}\right)e^{-i\left(\xi -\theta \right)\omega \tilde{\tau }}d\eta \left(\theta \right){\left(1,q_{1},q_{2}\right)}^{\text{T}}e^{i\xi \omega \tilde{\tau }}d\xi \\ =\bar{D}\left\{1+{\bar{q}}_{1}^{*}q_{1}+{\bar{q}}_{2}^{*}q_{2}-\int_{-1}^{0}\left(1,{\bar{q}}_{1}^{*},{\bar{q}}_{2}^{*}\right)\theta e^{i\theta \omega \tilde{\tau }}d\eta \left(\theta \right){\left(1,q_{1},q_{2}\right)}^{\text{T}}\right\}\\ =\bar{D}\left\{1+{\bar{q}}_{1}^{*}q_{1}+{\bar{q}}_{2}^{*}q_{2}+\tilde{\tau }q_{1}{\bar{q}}_{2}^{*}b\beta_{2}\left(\frac{\Lambda_{2}}{\mu_{2}}-V_{1}\right)e^{-i\omega \tilde{\tau }}-\tilde{\tau }q_{2}{\bar{q}}_{2}^{*}b\beta_{2}I_{1}e^{-i\omega \tilde{\tau }}\right\}\\ =1, \end{array}$$

thus

(79) $$\begin{array}{c} D=\frac{1}{1+q_{1}^{*}{\bar{q}}_{1}+q_{2}^{*}{\bar{q}}_{2}+\tilde{\tau }e^{i\omega \tilde{\tau }}\left({\bar{q}}_{1}q_{2}^{*}b\beta_{2}\frac{\mu_{2}V_{1}}{I_{1}}-{\bar{q}}_{2}q_{2}^{*}b\beta_{2}I_{1}\right)}. \end{array}$$

Next using the same notation as in [11] and a computation process similar to that in [18], we compute the center manifold *C*_0_ at *μ*=0.

Let *x*_*t*_ be the solution of (74).

Define

(80) zt=q∗,xt,Wt,θ=xtθ−2Reztqθ.

On the center manifold *C*_0_, we have

(81) $$\begin{array}{c} W\left(t,\theta \right)=W\left(z\left(t\right),\bar{z}\left(t\right),\theta \right), \end{array}$$

where

(82) $$\begin{array}{c} W\left(z\left(t\right),\bar{z}\left(t\right),\theta \right)=W_{20}\left(\theta \right)\frac{z^{2}}{2}+W_{11}\left(\theta \right)z\bar{z}+W_{02}\left(\theta \right)\frac{{\bar{z}}^{2}}{2}+\cdots . \end{array}$$

For the solution *x*_*t*_ ∈ *C*_0_ with *μ*=0, we can get

(83) z′t=iωτ˜z+q¯∗θ,f0,Wz,z¯,θ+2Rezqθ=iωτ˜z+q¯∗0f0,Wz,z¯,θ+2Rezq0=iωτ˜z+q¯∗0f0z,z¯≜iωτ˜z+gz,z¯,

where

(84) $$\begin{array}{c} g\left(z,\bar{z}\right)={\bar{q}}^{*}\left(0\right)f_{0}\left(z,\bar{z}\right)=g_{20}\frac{z^{2}}{2}+g_{11}z\bar{z}+g_{02}\frac{{\bar{z}}^{2}}{2}+g_{21}\frac{z^{2}\bar{z}}{2}+\cdots . \end{array}$$

From (80) and (81), we obtain that

(85) xtθ=x1tθ,x2tθ,x3tθT=Wt,θ+2Rezqθ=1,q1,q2Teiωτ˜θz+1,q¯1,q¯2Te−iωτ˜θz¯+W20θz22+W11θzz¯+W02θz¯22+⋯.

From (70) and (84),

(86) $$\begin{array}{c} g\left(z,\bar{z}\right)={\bar{q}}^{*}\left(0\right)f_{0}\left(z,\bar{z}\right)={\bar{q}}^{*}\left(0\right)f\left(0,x_{t}\right)\\ =\tilde{\tau }\bar{D}\left\{2\left(k_{1}+{\bar{q}}_{1}^{*}k_{2}+{\bar{q}}_{2}^{*}k_{3}\right)\frac{z^{2}}{2}+\left(l_{1}+{\bar{q}}_{1}^{*}l_{2}+{\bar{q}}_{2}^{*}l_{3}\right)z\bar{z}+2\left(m_{1}+{\bar{q}}_{1}^{*}m_{2}+{\bar{q}}_{2}^{*}m_{3}\right)\frac{{\bar{z}}^{2}}{2}+2\left(n_{1}+{\bar{q}}_{1}^{*}n_{2}+{\bar{q}}_{2}^{*}n_{3}\right)\frac{z^{2}\bar{z}}{2}\right\}. \end{array}$$

It follows

(87) $$\begin{array}{c} g_{20}=2\tilde{\tau }\bar{D}\left(k_{1}+{\bar{q}}_{1}^{*}k_{2}+{\bar{q}}_{2}^{*}k_{3}\right),\\ g_{11}=\tilde{\tau }\bar{D}\left(l_{1}+{\bar{q}}_{1}^{*}l_{2}+{\bar{q}}_{2}^{*}l_{3}\right),\\ g_{02}=2\tilde{\tau }\bar{D}\left(m_{1}+{\bar{q}}_{1}^{*}m_{2}+{\bar{q}}_{2}^{*}m_{3}\right),\\ g_{21}=2\tilde{\tau }\bar{D}\left(n_{1}+{\bar{q}}_{1}^{*}n_{2}+{\bar{q}}_{2}^{*}n_{3}\right), \end{array}$$

where

(88) $$\begin{array}{c} k_{1}=-\frac{b\beta_{1}q_{2}}{{\left(1+\alpha V_{1}\right)}^{2}}+\frac{\alpha b\beta_{1}S_{1}}{{\left(1+\alpha V_{1}\right)}^{3}}q_{2}^{2},\\ k_{2}=-\sigma b\beta_{1}q_{2}\left(1+q_{1}\right)+\frac{b\beta_{1}q_{2}}{{\left(1+\alpha V_{1}\right)}^{2}}-\frac{\alpha b\beta_{1}S_{1}}{{\left(1+\alpha V_{1}\right)}^{3}}q_{2}^{2},\\ k_{3}=-b\beta_{2}q_{1}q_{2}e^{-2i\omega \tilde{\tau }},\\ l_{1}=-\frac{b\beta_{1}}{{\left(1+\alpha V_{1}\right)}^{2}}\left({\bar{q}}_{2}+q_{2}\right)+\frac{2\alpha b\beta_{1}S_{1}}{{\left(1+\alpha V_{1}\right)}^{3}}q_{2}{\bar{q}}_{2},\\ l_{2}=-\sigma b\beta_{1}\left({\bar{q}}_{2}+q_{2}+q_{1}{\bar{q}}_{2}+{\bar{q}}_{1}q_{2}\right)+\frac{b\beta_{1}}{{\left(1+\alpha V_{1}\right)}^{2}}\left({\bar{q}}_{2}+q_{2}\right)-\frac{2\alpha b\beta_{1}S_{1}}{{\left(1+\alpha V_{1}\right)}^{3}}q_{2}{\bar{q}}_{2},\\ l_{3}=-b\beta_{2}\left(q_{1}{\bar{q}}_{2}+{\bar{q}}_{1}q_{2}\right),\\ m_{1}=-\frac{b\beta_{1}{\bar{q}}_{2}}{{\left(1+\alpha V_{1}\right)}^{2}}+\frac{\alpha b\beta_{1}S_{1}}{{\left(1+\alpha V_{1}\right)}^{3}}{\bar{q}}_{2}^{2},\\ m_{2}=-\sigma b\beta_{1}{\bar{q}}_{2}\left(1+{\bar{q}}_{1}\right)+\frac{b\beta_{1}{\bar{q}}_{2}}{{\left(1+\alpha V_{1}\right)}^{2}}-\frac{\alpha b\beta_{1}S_{1}}{{\left(1+\alpha V_{1}\right)}^{3}}{\bar{q}}_{2}^{2},\\ m_{3}=-b\beta_{2}{\bar{q}}_{1}{\bar{q}}_{2}e^{2i\omega \tilde{\tau }},\\ n_{1}=-\frac{b\beta_{1}}{{\left(1+\alpha V_{1}\right)}^{2}}\left(\frac{W_{20}^{\left(1\right)}\left(0\right)}{2}{\bar{q}}_{2}+\frac{W_{20}^{\left(3\right)}\left(0\right)}{2}+q_{2}W_{11}^{\left(1\right)}\left(0\right)+W_{11}^{\left(3\right)}\left(0\right)\right)+\frac{\alpha b\beta_{1}}{{\left(1+\alpha V_{1}\right)}^{3}}\left(q_{2}^{2}+2q_{2}{\bar{q}}_{2}\right)+\frac{\alpha b\beta_{1}S_{1}}{{\left(1+\alpha V_{1}\right)}^{3}}\left(W_{20}^{\left(3\right)}\left(0\right){\bar{q}}_{2}+2q_{2}W_{11}^{\left(3\right)}\left(0\right)\right)-\frac{3\alpha^{2}b\beta_{1}S_{1}}{{\left(1+\alpha V_{1}\right)}^{4}}q_{2}^{2}{\bar{q}}_{2},\\ n_{2}=-\sigma b\beta_{1}\left(\frac{W_{20}^{\left(1\right)}\left(0\right)}{2}{\bar{q}}_{2}+\frac{W_{20}^{\left(3\right)}\left(0\right)}{2}+q_{2}W_{11}^{\left(1\right)}\left(0\right)+W_{11}^{\left(3\right)}\left(0\right)+\frac{W_{20}^{\left(2\right)}\left(0\right)}{2}{\bar{q}}_{2}+\frac{W_{20}^{\left(3\right)}\left(0\right)}{2}{\bar{q}}_{1}+W_{11}^{\left(2\right)}\left(0\right)q_{2}+W_{11}^{\left(3\right)}\left(0\right)q_{1}\right)+\frac{b\beta_{1}}{{\left(1+\alpha V_{1}\right)}^{2}}\left(\frac{W_{20}^{\left(1\right)}\left(0\right)}{2}{\bar{q}}_{2}+\frac{W_{20}^{\left(3\right)}\left(0\right)}{2}+q_{2}W_{11}^{\left(1\right)}\left(0\right)+W_{11}^{\left(3\right)}\left(0\right)\right)-\frac{\alpha b\beta_{1}}{{\left(1+\alpha V_{1}\right)}^{3}}\left(q_{2}^{2}+2q_{2}{\bar{q}}_{2}\right)-\frac{\alpha b\beta_{1}S_{1}}{{\left(1+\alpha V_{1}\right)}^{3}}\left(W_{20}^{\left(3\right)}\left(0\right){\bar{q}}_{2}+2q_{2}W_{11}^{\left(3\right)}\left(0\right)\right)+\frac{3\alpha^{2}b\beta_{1}S_{1}}{{\left(1+\alpha V_{1}\right)}^{4}}q_{2}^{2}{\bar{q}}_{2},\\ n_{3}=-b\beta_{2}\left(\frac{W_{20}^{\left(2\right)}\left(-1\right)}{2}{\bar{q}}_{2}{\text{e}}^{i\omega \tilde{\tau }}+\frac{W_{20}^{\left(3\right)}\left(-1\right)}{2}{\bar{q}}_{1}{\text{e}}^{i\omega \tilde{\tau }}+W_{11}^{\left(2\right)}\left(-1\right)q_{2}{\text{e}}^{-i\omega \tilde{\tau }}+W_{11}^{\left(3\right)}\left(-1\right)q_{1}{\text{e}}^{-i\omega \tilde{\tau }}\right). \end{array}$$

By using (74) and (80), we can know

(89) W′=xt′−z′q−z¯′q¯=AW−2Req¯∗0f0qθ,θ∈−1,0,AW−2Req¯∗0f0qθ+f0,θ=0,≜AW+Hz,z¯,θ,

where

(90) $$\begin{array}{c} H\left(z,\bar{z},\theta \right)=H_{20}\left(\theta \right)\frac{z^{2}}{2}+H_{11}\left(\theta \right)z\bar{z}+H_{02}\left(\theta \right)\frac{{\bar{z}}^{2}}{2}+\cdots . \end{array}$$

From (81), (89), and (90), we can get

(91) $$\begin{array}{c} \left\{\begin{array}{c} \left(A-2i\omega \tilde{\tau }\right)W_{20}\left(\theta \right)=-H_{20}\left(\theta \right),\\ AW_{11}\left(\theta \right)=-H_{11}\left(\theta \right). \end{array}\right. \end{array}$$

From (89), (91), and the definition of *A*, we can calculate

(92) $$\begin{array}{c} W_{20}\left(\theta \right)=\frac{ig_{20}}{\omega \tilde{\tau }}q\left(0\right)e^{i\omega \tilde{\tau }\theta }+\frac{i{\bar{g}}_{02}}{3\omega \tilde{\tau }}\bar{q}\left(0\right)e^{-i\omega \tilde{\tau }\theta }+\Gamma_{1}e^{2i\omega \tilde{\tau }\theta }, \end{array}$$

(93) $$\begin{array}{c} W_{11}\left(\theta \right)=-\frac{ig_{11}}{\omega \tilde{\tau }}q\left(0\right)e^{i\omega \tilde{\tau }\theta }+\frac{i{\bar{g}}_{11}}{\omega \tilde{\tau }}\bar{q}\left(0\right)e^{-i\omega \tilde{\tau }\theta }+\Gamma_{2}, \end{array}$$

where Γ_*i*_=(Γ_*i*_^(1)^, Γ_*i*_^(2)^, Γ_*i*_^(3)^)^T^ ∈ *R* ^3^(*i*=1,2) are the three-dimensional vectors.

Now, we determine Γ_1_ and Γ_2_. By (91) and the definition of *A*, we have

(94) $$\begin{array}{c} \int_{-1}^{0}d\eta \left(\theta \right)W_{20}\left(\theta \right)=2i\omega \tilde{\tau }W_{20}\left(\theta \right)-H_{20}\left(\theta \right),\\ \int_{-1}^{0}d\eta \left(\theta \right)W_{11}\left(\theta \right)=-H_{11}\left(\theta \right), \end{array}$$

where *η*(*θ*)=*η*(*θ*, 0). By (89), when *θ*=0,

(95) $$\begin{array}{c} H\left(z,\bar{z},\theta \right)=-{\bar{q}}^{*}\left(0\right)f_{0}q\left(0\right)-q^{*}\left(0\right){\bar{f}}_{0}\bar{q}\left(0\right)+f_{0}\\ =-g\left(z,\bar{z}\right)q\left(0\right)-\bar{g}\left(z,\bar{z}\right)\bar{q}\left(0\right)+f_{0}. \end{array}$$

That leads to

(96) H200=−g20q0−g¯02q¯0+2τ˜−bβ11+αV12+αbβ1S11+αV13q22G0−bβ2q1q2e−2iωτ,H110=−g11q0−g¯11q¯0+2τ˜−bβ11+αV12Req2+αbβ1S11+αV13q2q¯2G1−bβ2Req1q¯2,

where

(97) G0=−σbβ1q2+q1q2+bβ1q21+αV12−αbβ1S11+αV13q22,G1=bβ11+αV12−σbβ1Req2−σbβ1Req1q¯2−αbβ1S11+αV13q2q¯2.

Since

(98) $$\begin{array}{c} \left(i\omega \tilde{\tau }I-\int_{-1}^{0}e^{i\theta \omega \tilde{\tau }}d\eta \left(\theta \right)\right)q\left(0\right)=0,\\ \left(-i\omega \tilde{\tau }I-\int_{-1}^{0}e^{-i\theta \omega \tilde{\tau }}d\eta \left(\theta \right)\right)\bar{q}\left(0\right)=0, \end{array}$$

we have

(99) $$\begin{array}{c} \left(2i\omega \tilde{\tau }I-\int_{-1}^{0}e^{2i\theta \omega \tilde{\tau }}d\eta \left(\theta \right)\right)E_{1}=2\tilde{\tau }\left(\begin{array}{c} -\frac{b\beta_{1}}{{\left(1+\alpha V_{1}\right)}^{2}}+\frac{\alpha b\beta_{1}S_{1}}{{\left(1+\alpha V_{1}\right)}^{3}}q_{2}^{2}\\ G_{0}\\ -b\beta_{2}q_{1}q_{2}e^{-2i\omega \tau } \end{array}\right). \end{array}$$

We can calculate

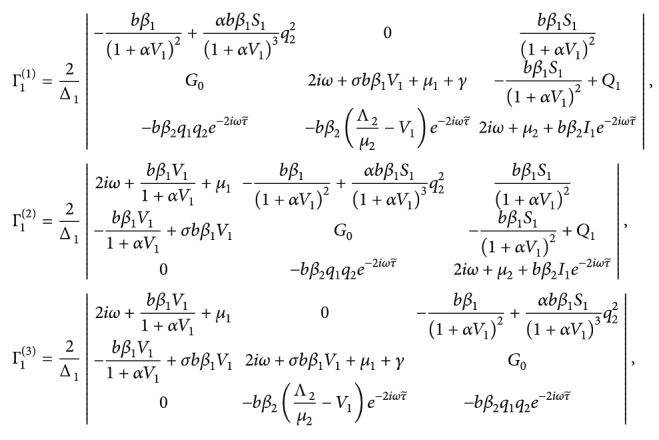
(100)where

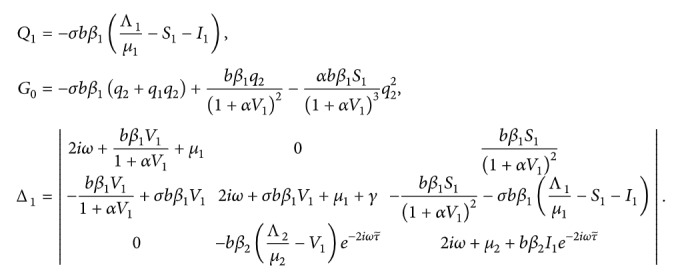
(101)


Similarly, we have

(102) ∫−10dηθΓ2=−2τ˜−bβ11+αV12Req2+αbβ1S11+αV13q22G1−bβ2Req1q¯2.

That leads to

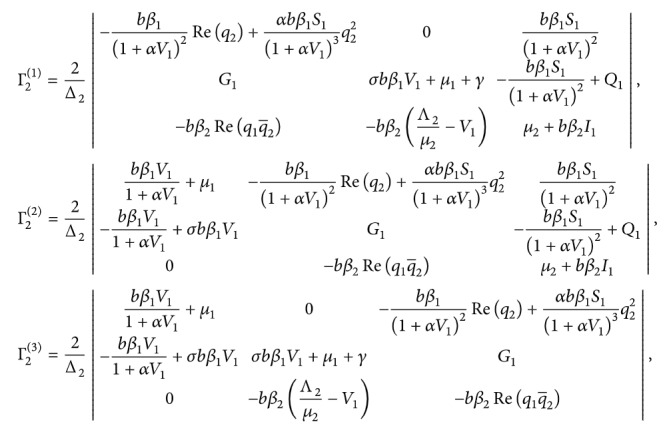
(103)where

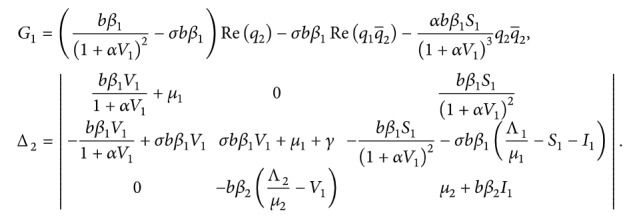
(104)


We can calculate *W*_20_(0) and *W*_11_(0) from (92) and (93). The above analysis shows that *g*_*ij*_ in (84) can be represented by using the parameters in the system (7). Thus, we can get the following equation:

(105) c10=i2ωτ˜g20g11−2g112−g0223+g212,μ2=−Rec10Reλ′τ˜,β2=2Rec10,T2=−Imc10+μ2Imλ′τ˜ωτ˜,

where the signs of *μ*_2_ determine the direction of the Hopf bifurcation of the system (7), the signs of *β*_2_ determine the stability of the bifurcated periodic solution, and *T*_2_ determine the period of the bifurcation periodic solution. We can summarize the following theorem:

**Theorem 7 .**

For the model (7), the direction and the stability of the periodic solution of Hopf bifurcation is determined by using (105) when $\tau =\tilde{\tau }$.

- If *μ*_2_ > 0, then the Hopf bifurcation is supercritical and the bifurcation periodic solution exists for $\tau >\tilde{\tau }$ in a $\tilde{\tau }$ neighborhood; if *μ*_2_ < 0, the Hopf bifurcation is subcritical and the bifurcation periodic solution exists for $\tau <\tilde{\tau }$ in a $\tilde{\tau }$ neighborhood.
- If *β*_2_ < 0, then the bifurcation periodic solution is stable; if *β*_2_ > 0, the bifurcation periodic solution is unstable.
- If *T*_2_ > 0, then the period of the bifurcation periodic solution increases; if *T*_2_ < 0, the period decreases.

## 6. Numerical Simulations

In this section, we use numerical simulations to illustrate our result about the existence of Hopf bifurcation.

The following parameters are selected: Λ_1_=8, *b*=0.29, *β*_1_=0.0033, *α*=0.06, *μ*_1_=0.0029, *σ*=0.48, *γ*=0.56, Λ_2_=9, *β*_2_=0.0059, and *μ*_2_=0.03. We can calculate that *R*_0_=80.2459 and *E*_1_=(444.9472, 450.8920, 288.7707).

**Figure 1:**
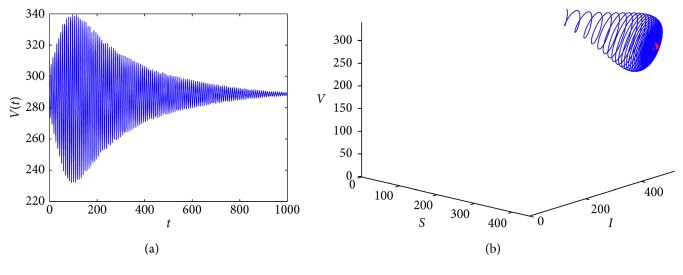
*E*_1_ is stable when *τ*=2.12.

**Figure 2:**
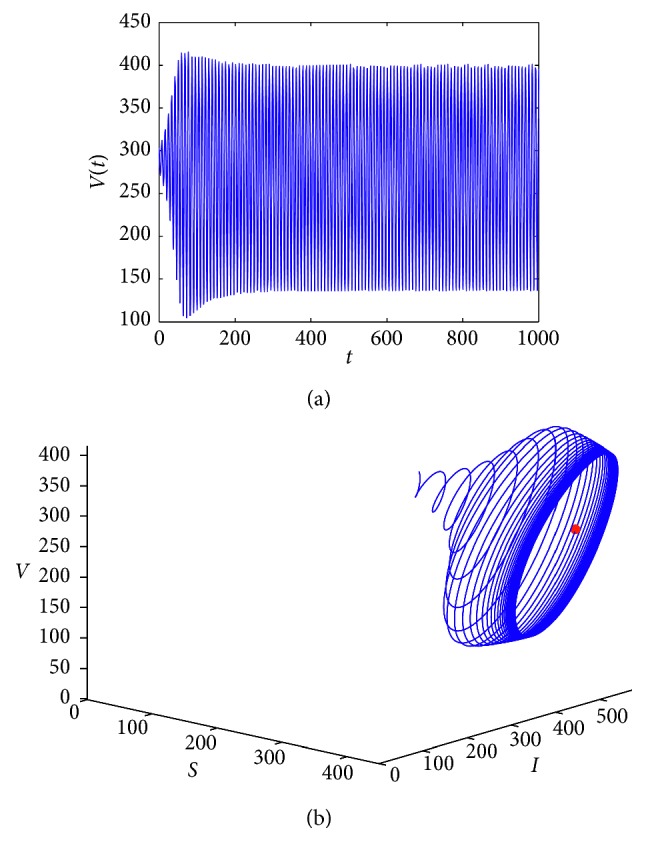
*E*_1_ undergoes Hopf bifurcation when *τ*=2.33.

Equation (37) has a positive root *x*=0.5779. We have *τ* ^*∗*^=2.1386. From Theorem 3.4, we know that the endemic equilibrium *E*_1_ is locally asymptotically stable for 0 < *τ* < *τ* ^*∗*^ (Figure 1), and system (7) undergoes a Hopf bifurcation at *E*_1_ when *τ*=*τ* ^*∗*^. At this time, we can calculate *c*_1_(0)=−2.3788 − 0.8427*i*, *μ*_2_=9.8850 > 0, *β*_2_=−4.7576 < 0, and *T*_2_=1.7152 > 0. According to Theorem 5.1, system (7) can produce a stable periodic solution, the Hopf bifurcation is supercritical at *τ* ^*∗*^, and the period of the bifurcation periodic solution increases (Figure 2).

## 7. Conclusions

In this paper, we discuss the dynamics of the vector-borne disease model with delay-saturated infection rate and reinfection. By calculation, we have the basic reproductive number *R*_0_. Through *R*_0_, we determined the existence of disease-free equilibrium *E*_0_ and the endemic equilibrium *E*_1_. According to the characteristic equation of the equilibrium points and using the Routh–Hurwitz criterion, we obtained that if *R*_0_ < 1 the disease-free equilibrium will be stable, and the endemic equilibrium is locally asymptotically stable if *R*_0_ > 1 and in the absence of time delay. Furthermore, by the fluctuation lemma and the limit theory, we analyzed the global stability of the disease-free equilibrium. We find that the time delay does not affect the stability of the boundary equilibrium but can change the stability of *E*_1_ and lead to the occurrence of Hopf bifurcation. Then by using the Nyquist criterion, we get the maximum length of delay to preserve stability. Next, we found that the conditions for determining the direction and stability of bifurcating periodic solutions. Finally, the correctness of the main conclusion is verified by numerical simulation.

## Data Availability

The data we selected is only to verify the correctness of the results. These data are not real data.
